# Precisely tuning the electronic states of organic polymer electrocatalysts via thiophene-based moieties for enhanced oxygen reduction reaction

**DOI:** 10.1016/j.isci.2025.112007

**Published:** 2025-02-12

**Authors:** Dongye Li, Binbin Wang, Kunpeng Zheng, Hongni Chen, Yali Xing, Yanzhi Xia, Xiaojing Long

**Affiliations:** 1 State Key Laboratory of Bio-fibers and Eco-textiles, Institute of Marine Biobased Materials, College of Materials Science and Engineering, Qingdao University, Qingdao 266071, P.R. China

**Keywords:** Catalysis, Electrochemistry, Materials science

## Abstract

Optimizing molecular structures in oxygen reduction reaction (ORR) is crucial for enhancing catalytic efficiency and stability, particularly with respect to the effective adsorption and conversion of reaction intermediates. Sulfur-containing heterocyclic compound thiophene can precisely modulate the electronic states and local charge densities, thereby fine-tuning the adsorption and reactivity of microporous polymers, yet, it remains a largely unexplored area. Herein, thiophene-based building blocks featuring diversified linkers into a phenyl-containing model Ph-CMP are developed, affording the thiophene-fused BPT-CMP and the thiophene-linked BCT-CMP. The electron density and adsorption capacity of the frameworks are well regulated through condensation and connecting modification, showing excellent half-wave potentials compared to the reversible hydrogen electrode, surpassing even most metal-free polymer electrocatalysts. Through theoretical calculations and experimental results, we have validated that the thiophene-fused skeleton (BPT-CMP) triggers the activation of thiophene units, with the exposed pentatomic heterocyclic carbon atom (site-3) serving as the catalytic active site.

## Highlights

• Thiophene-based moieties modulated conjugated polymers show excellent ORR performance
• The boron β-diketone units endow the polymer with excellent electron-accepting ability
• The thiophene-fused BPT-CMP promotes charge transfer and increases oxygen adsorption sites

## Graphical abstract

**Figure:**
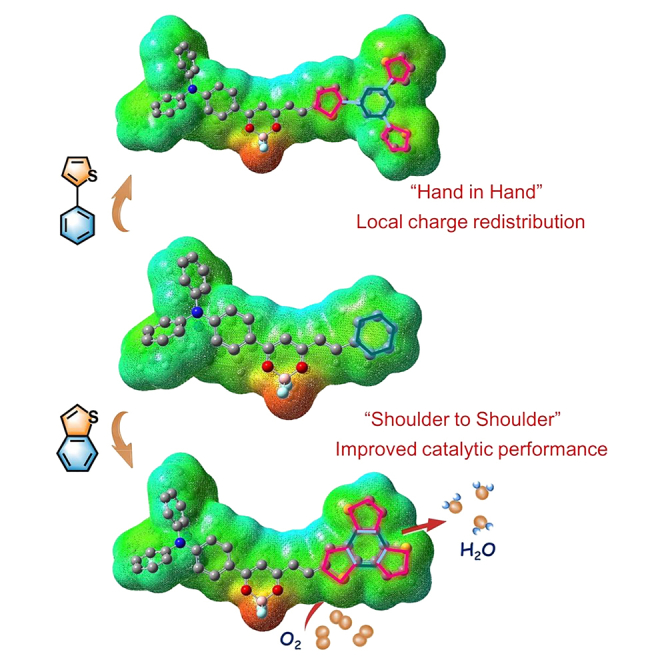

## Introduction

The oxygen reduction reaction (ORR) is crucial for electrochemical energy conversion, playing a pivotal role in energy conservation, emission reduction, and environmental pollution mitigation.^1^^,^^2^^,^^3^ Due to the high energy barrier inherent in the reaction process, efficient electrocatalysts are urgently needed to minimize their overpotential, resulting in increased energy efficiency, accelerated reaction kinetics, and reduced energy consumption in electrochemical energy conversion systems.^4^^,^^5^^,^^6^ Currently, platinum-based electrocatalysts are widely employed for ORR due to their exceptional catalytic activity and stability. However, their high cost, low crustal abundance, and limited methanol tolerance hinder further commercialization.^7^^,^^8^^,^^9^ To address these issues, the metal-free carbon-based electrocatalysts have been extensively explored as a promising alternative, focusing on charge redistribution as active centers to enhance fuel cell performance.^10^^,^^11^^,^^12^ While modulation engineering such as introducing structural defects and heteroatom doping through high-temperature pyrolysis, has proven effective,^13^^,^^14^^,^^15^ precisely designing metal-free carbon-based electrocatalysts with well-defined molecular structures and abundant active sites remains a significant challenge.

Conjugated microporous polymers (CMPs) represent a unique class of multifunctional porous materials featuring extended π-conjugated structures, permanent nanopores, high surface area, and diverse functional building blocks,^16^^,^^17^ making CMPs a cost-effective and ideal choice for applications optoelectronic materials,^18^^,^^19^ gas separation,^20^^,^^21^ catalysis,^22^^,^^23^ and drug delivery.^24^^,^^25^ Furthermore, CMPs possess abundant pore channels, controlled electronic environments, tailored site distribution, and remarkable thermal and chemical stability, rendering them highly promising for efficient and sustainable ORR catalysis.^26^^,^^27^^,^^28^ Compared to conventional building blocks in CMP frameworks, thiophene, a class of heterocyclic units, offers advantages such as distinctive electronic structures and excellent chemical stability.^29^^,^^30^^,^^31^ The rigid aromatic thiophene units, characterized by their peripheral π-electron topologies, enable exceptional intramolecular charge delocalization within the conjugated frameworks. Additionally, sulfur (S) atoms within the thiophene unit can induce charge redistribution and modify the electronic properties of adjacent carbon atoms. Consequently, CMPs containing thiophene exhibit numerous active sites and effective oxygen intermediate adsorption, enhancing the catalytic activity.^32^^,^^33^ Thus, incorporating thiophene units into precisely defined conjugated polymer frameworks is highly advantageous.

Biopolymers are crucial in synthesizing carbon materials due to their renewable and non-toxic properties. Given that the ocean covers three-quarters of the Earth’s surface, abundant sea resources represent a significant source of these materials.^34^^,^^35^ Alginate, an anionic and non-toxic polysaccharide naturally occurring in brown algae and certain bacteria, consists of α-L-guluronate (G) and β-D-mannuronate (M) residues linked by 1,4-glycosidic bonds. Additionally, alginate macromolecules are abundant in carboxyl and hydroxyl groups.^36^^,^^37^ During carbonization, these functional groups are released as carbon oxides and water, enabling the polymeric carbon structure to transform into carbon materials, which makes alginate an ideal precursor for producing porous carbon materials. Notably, by chelate divalent and trivalent metal ions, such as Ca^2+^, Co^2+^, Ni^2+^, Zn^2+^, and Fe^3+^, alginate can form an “egg-box” structure, which is advantageous for synthesizing metal-doped or metal-free three-dimensional carbon nanomaterials with multimodal pores. Compared to reduced graphene oxide and carbon nanotubes, this carbon-based material derived from marine biomass maintains high conductivity and supports the sustainable utilization of marine resources.

**Scheme 1:**
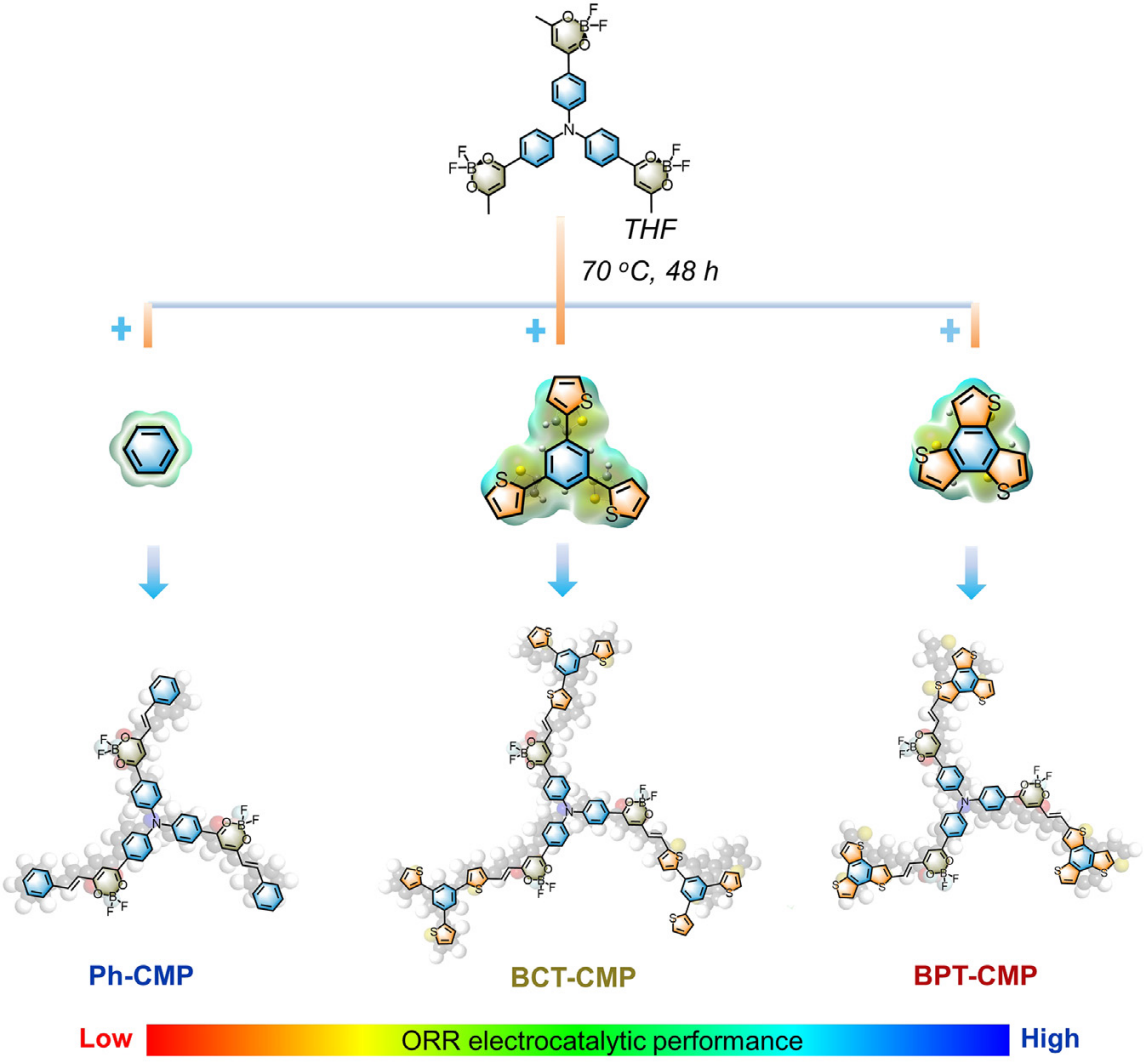
Synthetic route of the CMPs based on electron-deficient boron β-diketone units for Ph-CMP, BCT-CMP, and BPT-CMP

Herein, two thiophene-containing CMPs based on electron-deficient boron β-diketone units were designed and synthesized as ORR electrocatalysts (Scheme 1) by replacing the original phenyl units (Ph-CMP) with benzothiophene (BPT-CMP) and phenylthiophene units (BCT-CMP). The introduction of thiophene heterocycles breaks the uniform electron distribution of the molecular skeleton and achieves typical donor (D)-acceptor (A) properties. The thiophene linker regulating strategy further adjusts the electronic environment and enhances the interfacial active centers. The thiophene-fused BPT-CMP with biochar C_CA_ as a support skeleton exhibits superior ORR activity (half-wave potential of 0.75 V), surpassing most reported carbon-based electrocatalysts. Density functional theory (DFT) calculations indicate that the active adsorption sites of thiophene-based CMPs originate from the carbon atom (site-3) on the thiophene unit. Importantly, the thiophene linker strategy can modulate local charge redistribution, increase dipole moment, and enhance catalytic reaction kinetics.

## Results and discussion

### Synthesis and characterization

To implement the thiophene linker strategy, we intentionally used boron β-diketone monomers to construct a series of CMP catalysts (Figure S4C). The boron β-diketone based on boron-oxygen coordination bond (B←O) as a Lewis acid has excellent electron-accepting ability. In addition, the formation of stable six-membered boron-chelating rings in boron β-diketone units can suppress nonradiative energy dissipation through interconversion of O–H stretching mode between ketone and enol forms and further prolong π-conjugation of the skeleton to enhance electron-transporting ability.^38^ Meanwhile, the selected thiophene units with a delocalized π-electron system provide a rigid and planar structure, large surface area, and high-density activity, promoting intramolecular charge transfer and increasing oxygen adsorption sites in the polymer skeleton.^39^^,^^40^ Considering these factors, two CMPs were synthesized by reacting 1,3,5-triphenylamine with organic linkers 1,3,5-tri(benzothiophene)benzene (BPT) or 1,3,5-tri(2-thienyl)benzene (BCT) under solvothermal conditions via a Knoevenagel condensation reaction, denoted as BPT-CMP and BCT-CMP, respectively. The resulting CMPs demonstrate exceptional thermal stability, with decomposition temperatures (*T*_*d*_, 10% weight loss) exceeding 200°C (Figure S1A). Additionally, carbonized marine polysaccharide (C_CA_) is used as a support structure to improve the dispersion of the synthesized CMPs, offering the advantage of preserving high conductivity while promoting the sustainable use of marine resources.^41^

**Figure 1:**
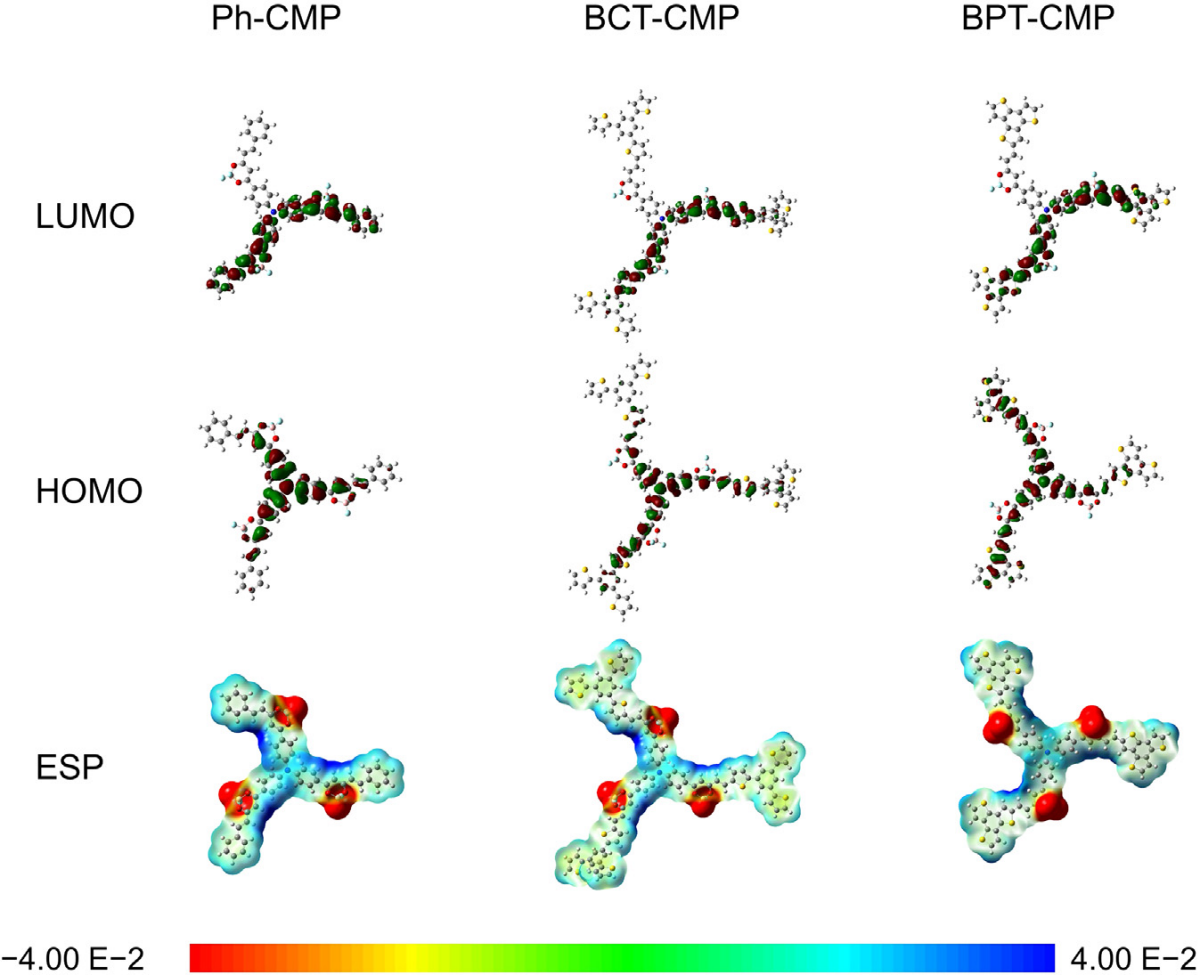
The calculated Kohn-Sham LUMOs, HOMOs, and electrostatic potential surface maps of Ph-CMP, BCT-CMP, and BPT-CMP

The influence of thiophene units on the electronic properties of metal-free organic molecules was investigated using DFT calculations. In metal-free carbon material systems, the inherently uniform charge distribution poses a challenge to achieving high catalytic efficiency. In Ph-CMP, negative charges are dispersed throughout the entire molecular skeleton (Figure 1),^42^ indicating effective electron delocalization. In contrast, the majority of charges in BPT-CMP and BCT-CMP are localized primarily around the thiophene units, disrupting the uniform charge distribution within the conjugated skeleton, and thereby regulating the electron density and adsorption capacity at specific active sites. Dipole moments, which serve as indicators of charge distribution and structural symmetry, are crucial for affecting molecular adsorption and reaction.^43^ DFT calculations indicate that thiophene-containing CMPs, BPT-CMP and BCT-CMP, exhibit dipole moments of 12.89 and 12.83 Debye, respectively, which are significantly higher than that of Ph-CMP (11.54 Debye). This observation suggests that the incorporation of thiophene units as building blocks enhances the molecular polarity of the CMPs, potentially facilitating intramolecular charge transfer processes.

**Figure 2:**
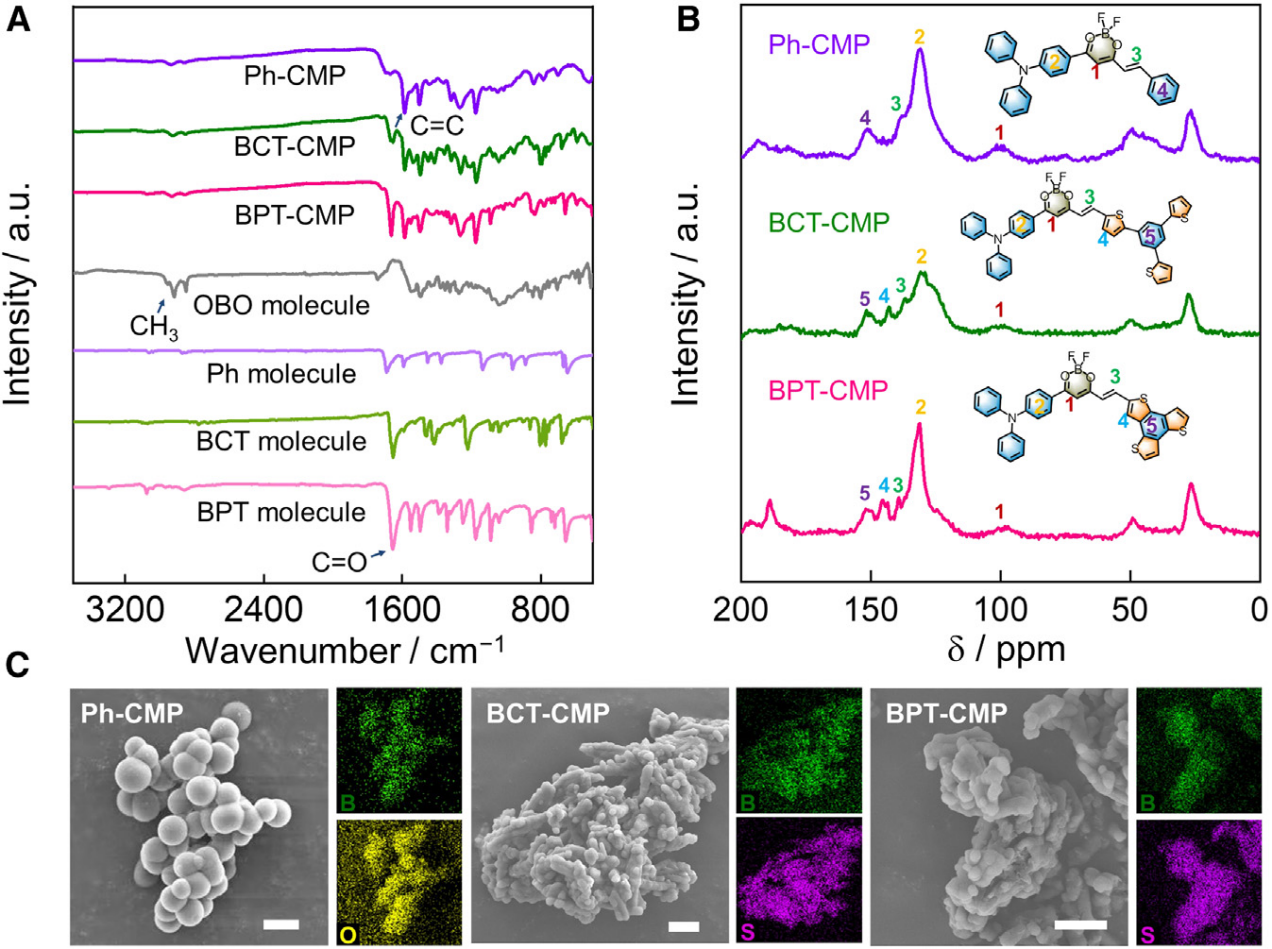
Structural and morphological characterization of Ph-CMP, BCT-CMP, and BPT-CMP (A) The FT-IR spectra of the three CMPs and the corresponding comonomers. (B) The solid-state ^13^C NMR spectrum of Ph-CMP, BCT-CMP, and BPT-CMP. (C) SEM image and corresponding EDS mappings of Ph-CMP, BCT-CMP, and BPT-CMP. Scale bars represent 2 μm.

Fourier transform infrared (FTIR) spectroscopy and solid-state ^13^C NMR spectrum were employed to characterize the chemical structures of the CMP frameworks. As depicted in Figure 2A, the FTIR spectra of these CMPs show a characteristic C=C stretching vibration mode at 1,588 cm^−1^ and the disappearance of the C=O stretching bands at 1,686 cm^−1^ from the thiophene comonomer. In addition, the absorption peak around 1,498 cm^−1^ corresponds to the deformation vibration of the C−S−C bond, indicating that the five-membered heterocyclic structure containing sulfur atoms has been successfully introduced into both BCT-CMP and BPT-CMP.^44^ The solid-state ^13^C NMR spectra of CMPs exhibit a prominent peak at 143 ppm (Figure 2B), corresponding to the thiophene heterocycle. Additionally, the vibration peak at 137 ppm is indicative of carbon atoms in the unsaturated C=C. These results confirm the successful Knoevenagel condensation reaction.^45^^,^^46^ To further assess the stability of these CMPs, they were exposed to solutions of 0.1 M KOH and 6.0 M KOH. FTIR spectroscopy analysis demonstrated that the CMP systems maintained their original molecular skeletons (Figures S1B–S1D), which is crucial for the ORR. Scanning electron microscopy (SEM) and transmission electron microscopy (TEM) revealed that these CMPs exhibit aggregated regular spherical and stick morphologies with micrometer-scale dimensions (Figures 2C and S2). Energy-dispersive X-ray spectroscopy (EDS) analysis confirmed the uniform distribution of O, S, and B atoms across the surface of CMPs, thereby excluding the influence of any residual reaction catalyst on the CMPs’ performance. Furthermore, the specific surface area and permanent porosity were determined using nitrogen (N_2_) adsorption-desorption isotherms at 77 K (Figures S3). The Brunauer-Emmett-Teller (BET) surface area of the thiophene-fused BPT-CMP is 8.02 m^2^ g^−1^ and the thiophene-linked BCT-CMP is 7.01 m^2^ g^−1^. The corresponding pore size adsorption curves indicate that the change of thiophene linkers did not significantly alter the microscopic porosity and surface area of the conjugated microporous polymers.

**Figure 3:**
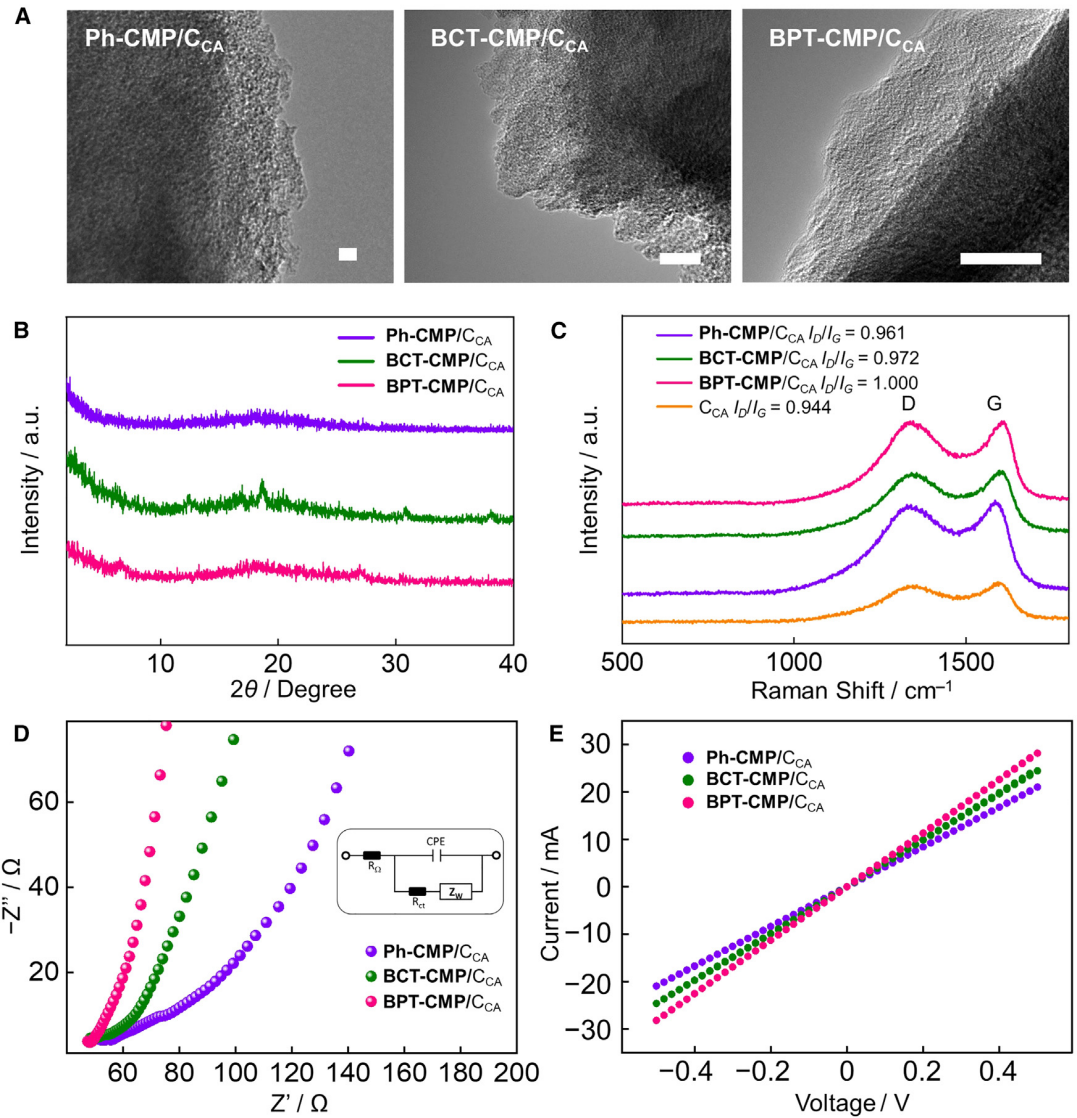
Structural and electrochemical performance characterization of Ph-CMP/C_CA_, BCT-CMP/C_CA_, and BPT-CMP/C_CA_ (A) TEM spectra of Ph-CMP/C_CA_, BCT-CMP/C_CA_, and BPT-CMP/C_CA_. Scale bars represent 10 nm. (B) The PXRD patterns of Ph-CMP/C_CA_, BCT-CMP/C_CA_, and BPT-CMP/C_CA_. (C) Raman spectra of Ph-CMP/C_CA_, BCT-CMP/C_CA_, BPT-CMP/C_CA_, and C_CA_. (D and E) (D) EIS spectra and (E) current (I)–voltage (V) curves of Ph-CMP/C_CA_, BCT-CMP/C_CA_, and BPT-CMP/C_CA_.

To gain a deeper understanding of the blending morphology of CMP heterostructures, TEM was utilized to observe the blended sample morphology (Figure 3A). The TEM images of the CMP/C_CA_ revealed a closely integrated state between the two components. Powder X-ray diffraction (PXRD) analysis certified the tight packing of the CMP/C_CA_ blends and verified the amorphous morphology of the CMPs (Figures 3B and S4A). Following the combination of C_CA_ with disordered CMPs, strong D-band and G-band intensities were observed in the Raman spectra of all CMP blends (Figure 3C). The intensity ratios of the D-band to G-band (*I*_*D*_/*I*_*G*_) for Ph-CMP/C_CA_, BCT-CMP/C_CA_, and BPT-CMP/C_CA_ were 0.961, 0.972, and 1.000, respectively, exceeding the 0.944 ratio of C_CA_, indicating that the incorporation of CMP increases the disorder within the blend. Furthermore, the electrochemical impedance spectroscopy (EIS) results for Ph-CMP/C_CA_ showed the highest arc radius and charge transfer resistance (R_ct_), suggesting that the inclusion of the thiophene unit aids in reducing charge transfer resistance (Figure 3D). Notably, the thiophene-fused BPT-CMP/C_CA_ exhibited the highest conductivity among the three CMP mixed systems (Figure 3E). The benzothiophene units, with the larger dipole moment, enhance intermolecular interactions and facilitate charge transfer, which is advantageous for the ORR process. The water contact angle test is a commonly employed method for evaluating the wettability of catalyst surfaces. By assessing the angle formed between a water droplet and the solid surface, the hydrophilicity or hydrophobicity of the material surface can be determined. The result revealed that all three CMPs exhibit hydrophilic properties (Figure S4B).^47^ Notably, the thiophene-fused BPT-CMP demonstrated exceptional hydrophilicity, absorbing the water droplet completely within just 0.54 s of contact. This remarkable hydrophilicity aids in reducing the interfacial resistance between the electrode and the electrolyte, thereby minimizing charge and mass transfer resistances and enhancing ORR catalytic performance.

**Figure 4:**
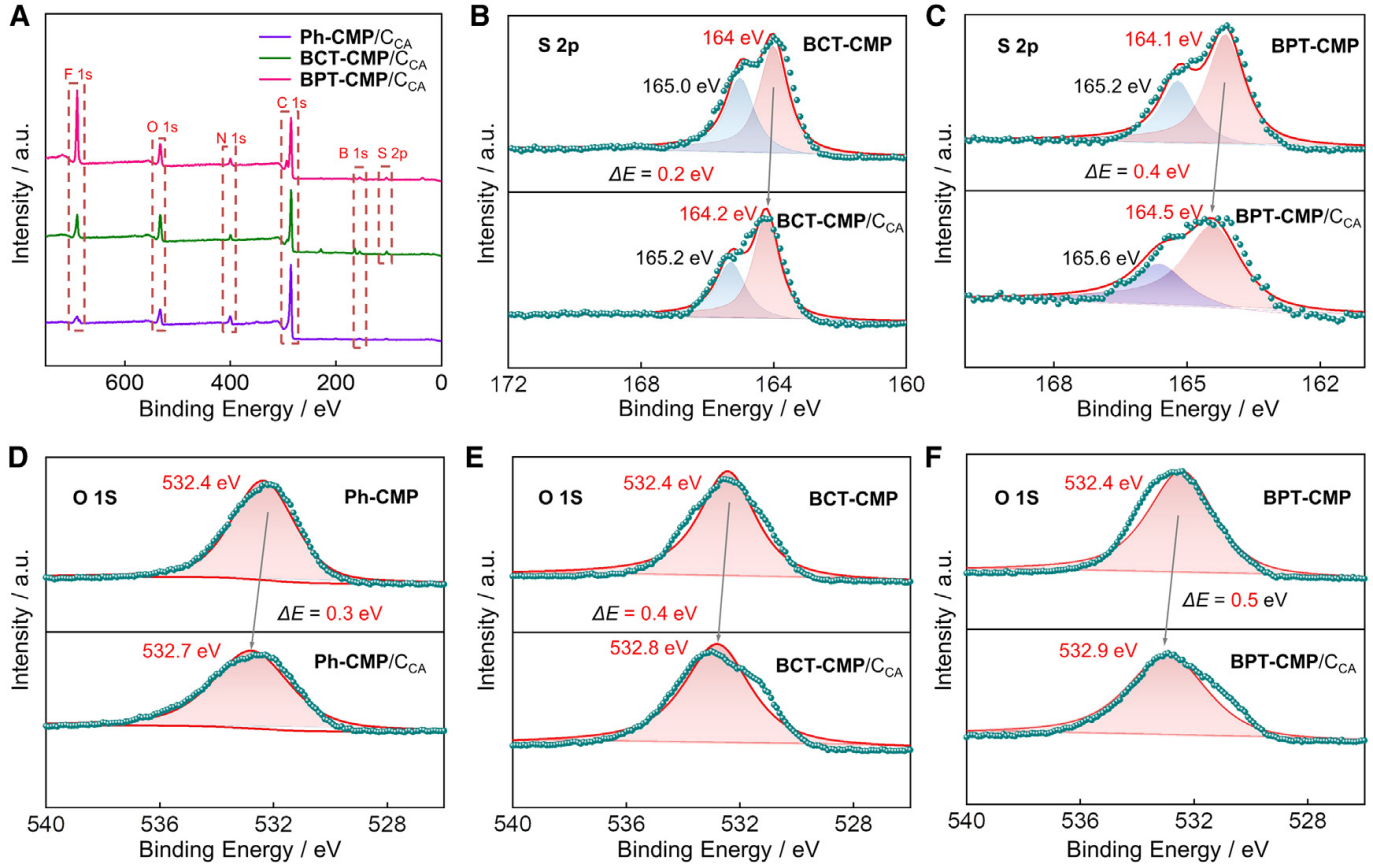
XPS spectra and fitted results (A) survey; S 2p of (B) BCT-CMP/C_CA_ and BCT-CMP. (C) BPT-CMP/C_CA_ and BPT-CMP; O 1s of (D) Ph-CMP/C_CA_ and Ph-CMP. (E) BCT-CMP/C_CA_ and BCT-CMP. (F) BPT-CMP/C_CA_ and BPT-CMP.

X-ray photoelectron spectroscopy (XPS) was employed to study the surface interaction between the prepared CMPs and C_CA_ (Figures 4A and S4D−S4F). The S 2p binding energy peaks of the two structures with sulfur heterocycles comprise S 2p 3/2 and S 2p 1/2 (Figures 4B and 4C). Notably, the combination of CMPs with C_CA_ results in the appearance of a new C 1s peak (293 eV), and causes the peaks of the elements to shift toward higher binding energy, attributable to electron transfer from CMPs to C_CA_. The S 2p binding energy of the thiophene-fused BPT-CMP differs by 0.4 eV from its blends, a difference larger than that of the thiophene-linked BCT-CMP (0.2 eV). Similar trends are observed in the O 1s binding energy profile (Figures 4D–4F). Specifically, the BPT-CMP and its blend systems display a larger binding energy difference of 0.5 eV for O 1s, compared to BCT-CMP (0.4 eV) and Ph-CMP (0.3 eV) systems, indicating a more significant electron transfer process. These results are primarily due to the large dipole moments, which facilitate interfacial charge transfer and enhance the kinetics of the catalytic reaction process of sulfur-containing molecules.

### Electrochemical performance

**Figure 5:**
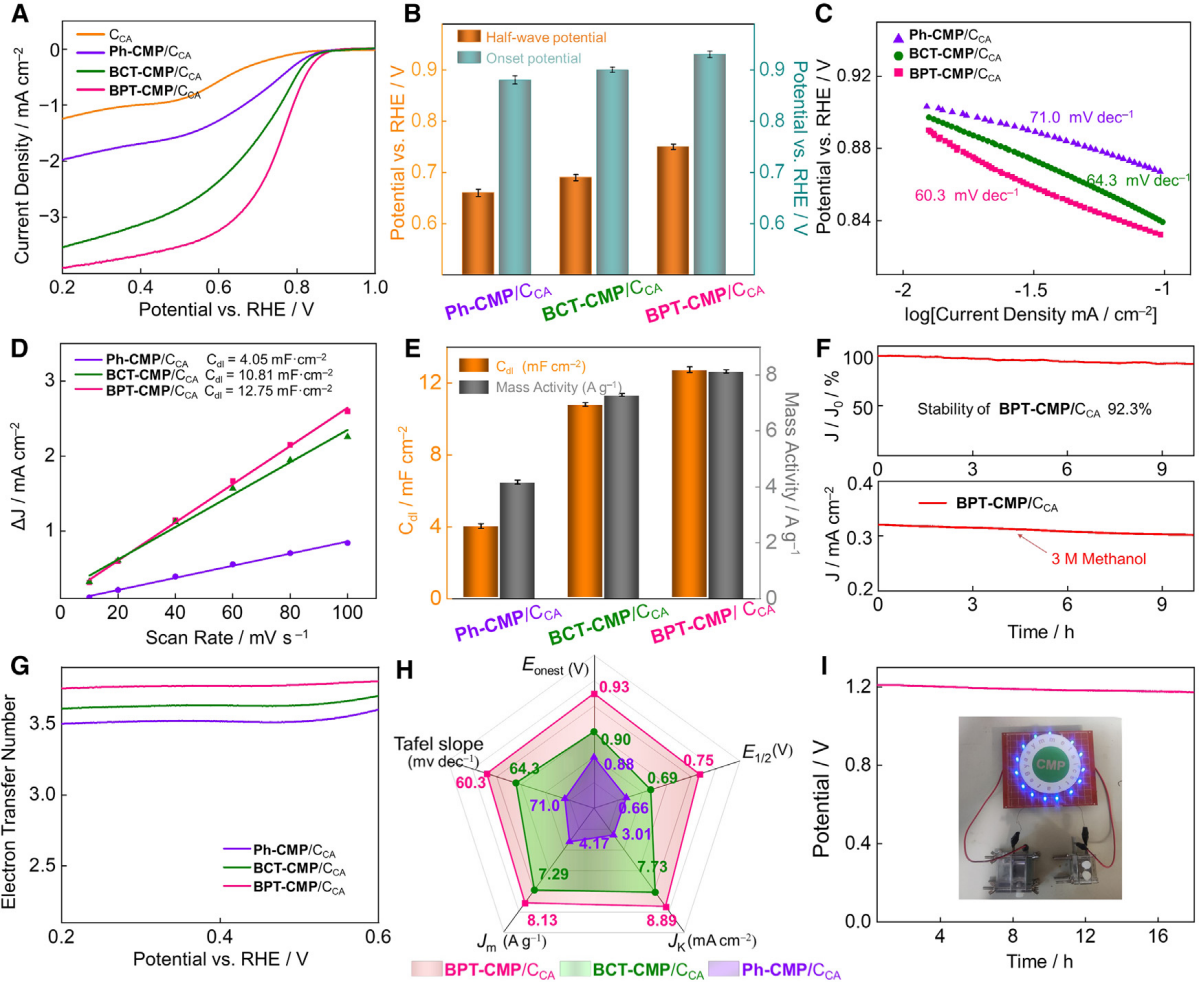
Electrocatalytic oxygen reduction reaction performance (A) LSV curves of Ph-CMP/C_CA_, BCT-CMP/C_CA_, BPT-CMP/C_CA_, and C_CA_. (B) Comparison of half-wave potential and onset potential for the ORR. (C) The corresponding Tafel plots (vs. RHE). (D) The capacitance values of Ph-CMP/C_CA_, BCT-CMP/C_CA_, and BPT-CMP/C_CA_. (E) Comparison of Cdl value and mass activity at 0.7 V versus RHE of Ph-CMP/C_CA_, BCT-CMP/C_CA_, and BPT-CMP/C_CA_. (F) Long-term stability test and CH_3_OH-poison effect on the i–t chronoamperometric response for Ph-CMP/C_CA_. (G) The electron-transfer number (n) is measured by RRDE at 1600 rpm. (H) Performance comparison of Ph-CMP/C_CA_, BCT-CMP/C_CA_, and BPT-CMP/C_CA_. (I) Galvanostatic discharge curve of BPT-CMP/C_CA_-based ZAB with 6.0 M KOH electrolyte and a photograph of an LED panel powered by two ZABs in series. (Data are represented as mean ± SEM. each experiment was independently tested three times; sample size *n* = 3; mean ± standard deviation (mean ± SD) was analyzed using Origin software; SD reflects the degree of dispersion among individuals samples; a small SD means that the value of the test is close to the average; the *p* value indicates significant differences: Ph-CMP/C_CA_ to BCT-CMP/C_CA_: *p* < 0.0001; BCT-CMP/C_CA_ to BPT-CMP/C_CA_: *p* < 0.0001; the statistical test was two-sided testing, the α-value was 0.05 and related *p* values were analyzed by a Student’s two-side test of GraphPad Prism software; *p* values less than 0.0001 indicate that the differences between Ph-CMP/C_CA_ to BCT-CMP/C_CA_ and BCT-CMP/C_CA_ to BPT-CMP/C_CA_ is particularly significant).

To further clarify the improvement of thiophene linker on the ORR catalytic activity, the CMPs/C_CA_ catalyst was tested in a 0.1 M KOH solution saturated with oxygen using a standard three-electrode system. The linear sweep voltammetry (LSV) curves at 1,600 rpm indicate that the onset potential (*E*_onset_) of the thiophene-fused BPT-CMP/C_CA_ is 0.93 V and its half-wave potential (*E*_1/2_) is 0.75 V, both significantly higher than those of BCT-CMP/C_CA_ (*E*_onset_ = 0.90 V, *E*_1/2_ = 0.69 V) and Ph-CMP/C_CA_ (*E*_onset_ = 0.88 V, *E*_1/2_ = 0.66 V) (Figures 5A, 5B, and S5A–S5C), demonstrating that the thiophene linker modulating strategy can dramatically enhance the ORR activity of the catalyst, surpassing even most metal-free polymer electrocatalysts (Figure S6C and Table S1). The thiophene-fused BPT-CMP/C_CA_ exhibited the lowest Tafel slope of 60.3 mV dec⁻^1^, indicating faster kinetics compared to thiophene-linked BCT-CMP/C_CA_ (64.3 mV dec⁻^1^) and model Ph-CMP/C_CA_ (71.0 mV dec⁻^1^) (Figure 5C). This result is also consistent with the kinetic current density (*J*_k_) measurements (3.01 mA cm⁻^2^ for Ph-CMP/C_CA_, 7.73 mA cm⁻^2^ for BCT-CMP/C_CA_, and 8.89 mA cm⁻^2^ for BPT-CMP/C_CA_) (Figure 5H).^48^

The electrochemical double-layer capacitance (C_dl_) was determined using cyclic voltammetry curves, providing a quantitative measure to evaluate the electrochemical active surface area (ECSA) of the materials (Figure 5D). As a result, the thiophene-containing CMP catalysts exhibited higher C_dl_ values (12.75 mF cm^−2^ for BPT-CMP/C_CA_ and 10.81 mF cm^−2^ for BCT-CMP/C_CA_) compared to phenyl-containing catalytic structure (4.05 mF cm⁻^2^ for Ph-CMP/C_CA_) (Figures S5D–S5F). This trend was also observed in mass activity measured at 0.70 V vs. reversible hydrogen electrode (RHE) (8.13 A g^−1^ for BPT-CMP/C_CA_, 7.29 A g^−1^ for BCT-CMP/C_CA_, and 4.17 A g^−1^ for Ph-CMP/C_CA_) (Figure 5E). The C_dl_ and mass activity results further demonstrate that, due to non-uniform charge distribution, benzothiophene units provide superior activity density. The BPT-CMP/C_CA_ demonstrated excellent long-term stability, with only a 7.7% loss in current density after 9 h in 0.1 M KOH electrolyte, demonstrating outstanding electrochemical stability (Figure 5F). Moreover, the addition of a 3 M CH_3_OH solution did not significantly affect the current density, demonstrating excellent methanol resistance of BPT-CMP/C_CA_. After conducting stability tests, we collected the materials and performed infrared spectroscopy on them. Essentially, there were no changes observed between the obtained infrared spectra and the initial infrared spectra (Figures S1B–S1D). The electron transfer number (n) of the catalyst was gauged in an oxygen-saturated 0.1 M KOH solution using a rotating ring disk electrode. In the range of 0.2–0.6 V, the electron transfer number of BPT-CMP/C_CA_ ranged from 3.75 to 3.80, suggesting an approximate four-electron transfer process (Figures 5G and S6A). The number of transferred electrons calculated by Koutecky-Levich plots at 0.2 V (vs. RHE) also follows the corresponding pattern (Figure S6B) The air diffusion layer with three mixed load systems was used as the air electrode for zinc-air batteries (ZABs) (Figures 5I and S7A–S7C).^49^^,^^50^ The BPT-CMP/C_CA_-based ZAB achieved a current density of 123.1 mA cm^−2^ at 1.0 V, and a maximum power density of 0.219 W cm^−2^ at 0.57 V, which exhibited excellent rate performance and voltage stability. At a current density of 5 mA cm^−2^, the voltage of the BPT-CMP/C_CA_-based ZAB maintained 1.18 V after 12 h of constant discharge, and two series-connected BPT-CMP/C_CA_-based ZABs successfully powered a light-emitting diode.

### DFT calculations

**Figure 6:**
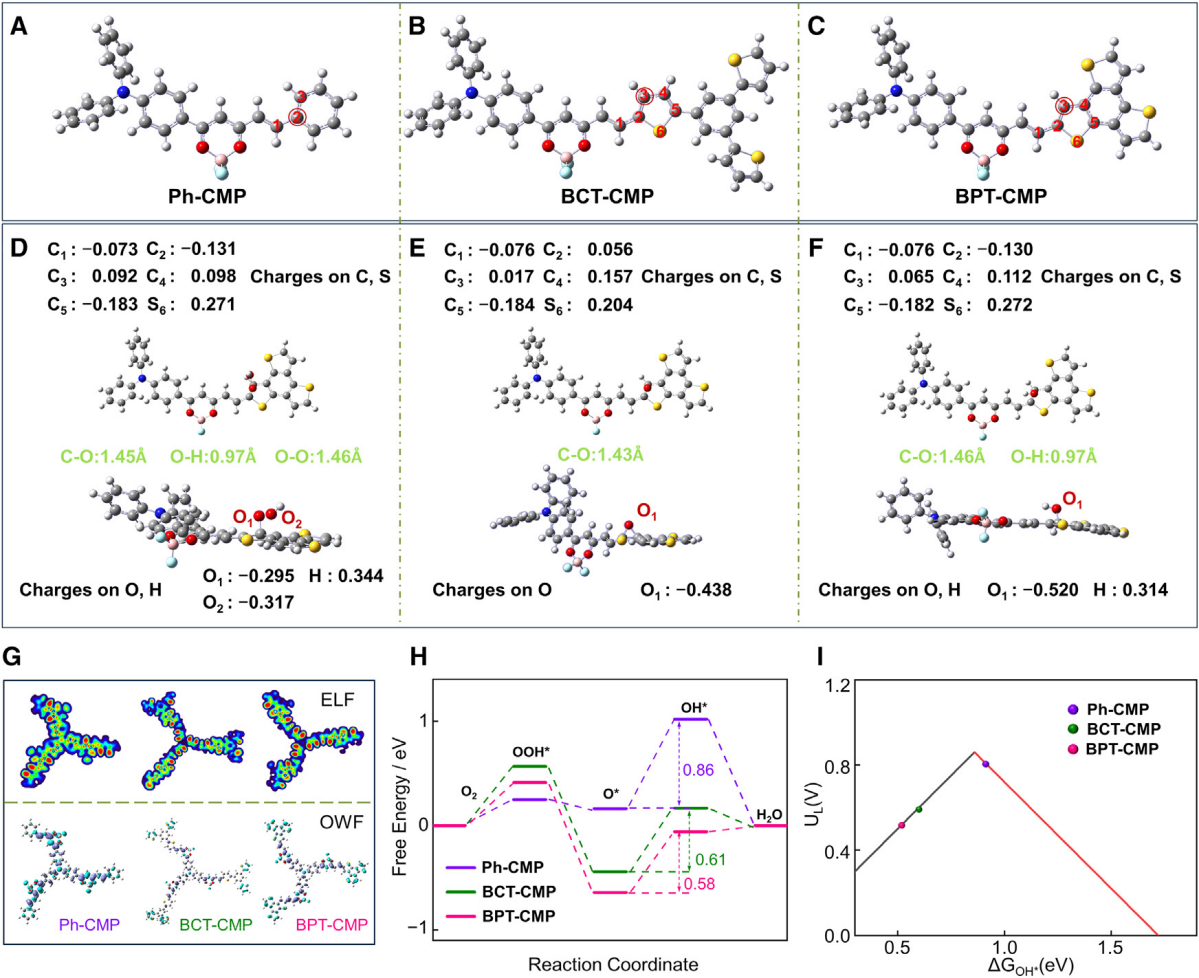
DFT calculations of Ph-CMP, BCT-CMP, and BPT-CMP (A–C) Optimized Ph-CMP, BCT-CMP, and BPT-CMP model structures. The gray, blue, yellow, and white balls represent C, N, S, and H atoms, respectively. (D–F) The optimized geometrical structures of stationary points along the reaction pathway of BPT-CMP catalyzing ORR reaction, (D) OOH∗, (E) O∗, (F) OH∗, along with the main geometrical parameters and the Mulliken charges on main atoms. (G) Electron localization function (Up) and orbit weight fukui function (Down, Blue, and green represent the positive and negative parts, respectively). (H) Free energy diagrams for Ph-CMP, BCT-CMP, and BPT-CMP. (I) Activity volcanoes for the 4e^−^ oxygen reduction reaction.

To gain deeper insights into the distinct catalytic performance of the thiophene-modulated CMPs, DFT calculations were employed to identify specific active sites and clarify the mechanisms in their catalytic reaction process. Three theoretical models were constructed, each representing the repeating units of Ph-CMP, BCT-CMP, and BPT-CMP (Figures 6A−6C), respectively.^51^ During the calculations, we focused on BPT-CMP and evaluated its performance in catalyzing the ORR by calculating Mulliken charges and geometric parameters for key atoms along the reaction pathway (Figures 6D and 6F). The oxygen atom in O₂ interacts with the sulfur atom in BPT-CMP. Initially, the C−O bond length is measured at 1.45 Å. Subsequently, electron transfer occurs, leading to the formation of O∗ and shortening the C−O bond to 1.43 Å, thereby enhancing charge transfer between BPT-CMP and O∗. As the reaction advances further, the C−O bond length increases again to 1.46 Å, facilitating the progression of the ORR catalytic process.

Electrophilic sites were predicted by Fukui function to identify active sites. It is worth noting that the highest distribution of the isosurface map in the thiophene-fused BPT-CMP will be more conducive to the adsorption of electrophilic oxygen intermediates (Figure 6G).^52^^,^^53^^,^^54^ The electron location function (ELF) further reveals that the thiophene-fused BCT-CMP and the thiophene-linked BPT-CMP enhanced larger localization around the S atom region, promoting the ORR catalytic reaction. The free-energy diagrams (ΔG) were calculated to identify overpotentials.^55^ The calculations indicate that the carbon atom at the 3-position of thiophene unit in BPT-CMP acts as an active site, with an overpotential of 0.58 eV (Figure 6H and Tables S2−S4). This value is lower compared to those of BCT-CMP (0.61 eV at site-3) and Ph-CMP (0.86 eV at site-3). Furthermore, when BPT-CMP adsorbs various reaction intermediates, it undergoes structural changes and surface electrostatic potential shifts that are in agreement with Gibbs free energy calculations, conforming to the four-electron reaction pathway of the ORR (Figure S7D). Additionally, the volcano plot exhibits a correlation between the overpotentials of reaction sites on Ph-CMP, BCT-CMP, and BPT-CMP (Figure 6I). These results not only identify the active sites in BPT-CMP during the catalytic ORR process but also offer valuable theoretical insights for the design and optimization of novel and high-efficiency catalysts.

### Conclusions

In summary, we presented a thiophene linker modulating approach to synthesize metal-free CMP electrocatalysts featuring thiophene-fused and thiophene-linked bonding modes with unique boron β-diketone molecules. The resultant CMPs exhibited precisely tuned charge redistribution and exceptional conductivity. Notably, the thiophene-fused BPT-CMP displayed superior catalytic activity toward ORR. Based on DFT calculations and experimental analysis, the sulfur-containing heterocyclic thiophene with diversified linkers in CMPs effectively adjusts charge distribution, increases molecular dipole moments, and optimizes oxygen adsorption energy. Furthermore, these heterocyclic structures efficiently activate the neighboring sp-hybridized carbon atom (site-3) as an active site, thereby lowering the free energy, which is crucial for the catalytic reaction process. This work deepens our understanding of metal-free CMPs and demonstrates that the precise control of thiophene heterocycle linker offers a novel strategy to explore the intricate relationship between catalyst structure and catalytic activity.

### Limitations of the study

The limitation of this study is that, in terms of performance alone, the prepared metal-free organic molecules still lags behind Pt-based materials. Therefore, it is still necessary to conduct more structural design and synthesis in the future to improve the ORR performance of organic catalysts.

## Resource availability

### Lead contact

Further information and requests for resources and reagents should be directed to and will be fulfilled by the lead contact, Xiaojing Long (longxj@qdu.edu.cn).

### Materials availability

This study did not generate new unique reagents.

### Data and code availability

• All data reported in this article will be shared by the lead contact upon request.
• This article does not report the original code.
• Any additional information required to reanalyze the data reported in this article is available from the lead contact upon request.

## Acknowledgments

The authors are grateful for the financial support from the 10.13039/501100001809National Natural Science Foundation of China (nos. 22375111 and 22075157), the Natural Science Foundation and Youth Innovation Team Project of Shandong Province, China (nos. ZR2024JQ037, 2021KJ018, and ZR2021YQ08), and the Taishan Scholars Program (no. tsqn201909090).

## Author contributions

D.L. and B.W. contributed equally; conceptualization, B.W. and X.L.; data curation, Yali Xing and H.C.; writing–original draft, D.L. and K.Z.; writing–review and editing, X.L and B.W.; resources, K.Z.; supervision, Yanzhi Xia.

## Declaration of interests

The authors declare no competing interests.

## STAR★Methods

### Key resources table

**Table:** 

REAGENT or RESOURCE	SOURCE	IDENTIFIER
**Chemicals, peptides, and recombinant proteins**		

Triphenylamine	Shanghai Macklin Biochemical Technology Co., Ltd	CAS#603-34-9
Boron trifluoride acetic acid complex	Shanghai Macklin Biochemical Technology Co., Ltd	CAS#373-61-5
1,3,5-Benzenetricarboxaldehyde	Shanghai Macklin Biochemical Technology Co., Ltd	CAS#3163-76-6
Benzene-1,3,5-triyl tris(thiophene-2-carbaldehyde)	Shanghai Haohong Biomedical Technology Co., Ltd	CAS#2125450-22-6
Benzo[1,2-b:3,4-b':5,6-b'']trithiophene-2,5,8-tricarbaldehyde	Shanghai Haohong Biomedical Technology Co., Ltd	CAS#2243590-42-1
Sodium alginate	Shanghai Macklin Biochemical Technology Co., Ltd	CAS# 9005-38-3

**Software and algorithms**		

Origin	Smith et al.	https://www.originlab.com
GraphPad Prism	La Jolla	RRID:SCR 002798
GaussView	Semichem,Inc.	https://gaussian.com

### Method details

#### Synthesis

All reactions were conducted under an argon atmosphere. Tetrahydrofuran was dried using sodium or calcium hydroxide before use. Other commercially available solvents and reagents were used without further purification unless stated otherwise.

Triphenylamine Difluoroboronate Monomer: Triphenylamine (100 mg, 0.408 mmol), acetic anhydride (1.8 g, 18.36 mmol), and boron trifluoride acetic acid complex (1.15 g, 6.12 mmol) were added to a three-neck round-bottom flask. The mixture was degassed for 10 minutes before introducing argon. Dry THF was subsequently added, and the reaction was conducted at 45°C for 12 hours. Upon completion, the reaction mixture was extracted with dichloromethane, and the organic phase was purified by column chromatography. The product was collected and dried under vacuum (83% yield). ^1^H NMR (400 MHz, CDCl3, 25°C) *δ* 8.05 (d, *J* = 8.9 Hz, 2H), 7.24 (d, *J* = 8.9 Hz, 2H), 6.51 (s, 1H), 2.43 (s, 3H).

##### Ph-CMP

Triphenylamine difluoroboronate derivative (100.1 mg, 0.156 mmol), 1,3,5-Benzenetricarboxaldehyde (25.3 mg, 0.156 mmol), and piperidine (0.1 mg, 0.001 mmol) were added to a three-neck round-bottom flask. The mixture was degassed for 10 minutes and then backfilled with argon. Using a syringe, 6 mL of dry THF was added to the flask. The reaction mixture was stirred at 120°C for 48 hours. After cooling to room temperature, the mixture was filtered and washed with dichloromethane, water, acetone, and methanol, followed by Soxhlet extraction with THF for purification. Finally, the orange powdery polymer **Ph-CMP** was obtained after drying in a 60°C oven for 12 hours (83% yield).

##### BCT-CMP

The **BCT-CMP** was synthesized from benzene-1,3,5-triyl tris(thiophene-2-carbaldehyde) and triphenylamine difluoroboronate derivative following the synthesis procedure of **Ph-CMP** (75% yield).

##### BPT-CMP

The **BPT-CMP** was synthesized from benzo[1,2-b:3,4-b':5,6-b'']trithiophene-2,5,8-tricarbaldehyde and triphenylamine difluoroboronate derivative following the synthesis procedure of **Ph-CMP** (79% yield).

Preparation of carbonized calcium alginate (C_CA_). The as-prepared sodium alginate solution with a mass fraction of 4% was added dropwise to the 0.1 M calcium chloride solution. After 8 hours of stirring, filtering the fully cross-linked microspheres, then washed with deionized water, dried and placed in a tubular furnace, heated at 800°C for two hours under nitrogen atmosphere at a heating rate of 5°C min^−1^, and then naturally cooled to room temperature. The carbonized sample was soaked in 1 M hydrochloric acid and then washed with deionized water until neutral. After drying at 110°C for 6 hours, it can be finally ground into powder for later use.

Preparation of CMPs/C_CA_: 3 mg of CMPs and 3 mg of carbonized calcium alginate are put in a mortar, grinding for 30 minutes to mix them uniformly.

#### Characterizations

^1^H and solid-state ^13^C NMR spectra were tested with a Bruker AV-400 spectrometer in CDCl_3_ at 25°C. Chemical shifts were reported in *δ* ppm using CHCl_3_ (7.26 ppm) for ^1^H NMR. Thermal analysis was performed on a TG 209 instrument under nitrogen flow at a heating rate of 10°C min^–1^. The morphologies and structures of the samples were characterized by using field emission scanning electron microscopy (FESEM; JSM-7001F, JEOL, Tokyo, Japan) with an energy dispersive X-ray spectrometer (EDS) and the transmission electron microscopy (TEM; JEM–1011; JEOL Co., Japan) operated at an accelerating voltage of 100 kV. The molecular packing was investigated by powder X-ray diffraction (PXRD). PXRD data were collected on a Bruker D8. Empyrean powder diffractometer using a Cu Kα source (λ = 1.5418 Å) over the range of 2θ = 2.0–40.0° with a step size of 0.02° and 2 s per step. The current (I)-voltage (V) curves were measured by a source Meter (2612B, Keithley) at room temperature. The X-ray photoelectron spectrometry (XPS) was conducted on an XPS instrument (Axis Supra). FT-IR data were performed on a Nicolet iS50 FT-IR spectrometer (Thermo Fisher Scientific Co.). Raman spectroscopy was obtained on a Thermo Scientific DXR2 Raman spectrometer. The contact angle was measured by the Attention Optical Contact Angle measuring instrument theta (Sweden baioulin Technology Co., Ltd). To estimate pore size distributions, nonlocal density functional theory (NLDFT) was applied to analyze the N_2_ isotherm based on the model of N_2_@77K on carbon with slit pores and the method of non-negative regularization.

#### Electrochemical performance testing

All ORR measurements were conducted in Pine Research Instrumentation at ambient temperature in a CHI 760E electrochemical workstation (CH Instruments Co., China). For the electrochemical test, 6 mg of the catalyst was dispersed in the mixture of 80 μL of Nafion solution (5 wt%), 260 μL of ethanol, and 260 μL distilled water. Then homogenous catalyst ink was obtained by an ultrasonic disperse method. 6 μL of the solution was loaded onto the glass carbon electrode (0.126 cm^2^). The catalyst mass loading is 0.48 mg cm^‒2^. Measurements were performed in a three-electrode equipped with a graphite rod counter electrode and Ag/AgCl reference electrode. The electrolytic cell radius is 3.5 cm and the electrolytic cell height is 6 cm. Cyclic voltammetry (CV) experiments with a sweep rate of 50 mV s^−1^ were recorded in the potential range of 0 to −1.0 V vs. Ag/AgCl. Linear sweep voltammetry (LSV) was performed in N_2_-saturated or O_2_-saturated 0.1 M aq KOH. Current-voltage curves were recorded at a scan rate of 10 mV s^−1^ under various electrode rotation rates (400, 625, 900, 1225, 1600, 2025, and 2500 rpm, respectively). The current density was normalized to the geometrical area and the measured potentials vs. Ag/AgCl were converted to a reversible hydrogen electrode (RHE) scale according to the following equation: E_RHE_ = E_Ag/AgCl_ + 0.059 ∗ pH + 0.1976.

Rotating ring-disk electrode (RRDE) was carried out in an O_2_-saturated 0.1 M KOH electrolyte at room temperature using a CHI760E electrochemical workstation equipped with a modulated speed electrode rotator (RRDE-3A).

A long-term stability test was conducted by measuring the current changes of the catalyst at a fixed potential of 0.6 V (vs RHE) at a rotation speed of 1600 rpm in an O_2_-saturated electrolyte using CHI 760 E electrochemical workstation.

Electrochemical Measurements for the Zinc-Air Battery: the air electrode was prepared by uniformly coating the as-prepared catalyst ink onto carbon paper and then drying it at 60°C for 1 h. The mass loading was 1.5 mg cm^−2^. A zinc plate was used as the anode. Both electrodes were assembled into a home-built electrochemical battery with the electrolyte being a 6 M KOH electrolyte.

#### Computational details

B3LYP hybrid density functional theory of Gaussian 09 was employed with a basis set of 6-31G (d, p). Gibbs free energy (ΔG) diagrams and charge density were calculated by Vienna Ab initio Simulation Package (VASP) code. The exchange-correlation energy was modelled by using Perdew-Burke-Ernzerhof (PBE) functional within the generalized gradient approximation (GGA). The projector-augmented wave (PAW) pseudo-potentials were used to describe ionic cores. The cutoff energy of 500 eV was adopted. The convergence threshold for the iteration in the self-consistent field (SCF) was set to be 10^–4^ eV. Denser k-points (1×1×1) were used for the electronic structure calculations. A large vacuum slab of 20 Å was inserted in z-direction for surface isolation to prevent interaction between two neighbouring surfaces. The overall ORR in an acid environment can be written as follows:

$$O_{2}\left(g\right)+H_{2}O\left(l\right)+e^{-}+*\to OOH^{*}+OH^{-}$$

$$\text{OO}H^{*}+e^{-}\to O^{*}+OH^{-}$$

$$O^{*}+H2O\left(l\right)+e^{-}\to OH^{*}+OH^{-}$$

$$OH^{*}+e^{-}\to OH^{-}+*$$

For each elementary step, the Gibbs reaction free energy ΔG is defined as the difference between free energies of the initial and final states and is given by the expression

$$\Delta G=\Delta E+\Delta \text{ZPE}-T\Delta S+{\Delta G}_{U}+{\Delta G}_{PH}$$

where ΔE is the reaction energy of reactant and product molecules adsorbed on catalyst surface, obtained from DFT calculations; ΔZPE and ΔS are the changes in zero-point energies and entropy due to the reaction. Each step of ΔG was obtained as follows:

(Equation 1) $${\Delta G}_{a}={\Delta G}_{{\text{OOH}}^{*}}-eU+{\Delta G}_{H^{+}}-4.92\;\text{eV}$$

(Equation 2) $${\Delta G}_{b}={\Delta G}_{O^{*}}-{\Delta G}_{{\text{OOH}}^{*}}-eU+{\Delta G}_{H^{+}}$$

(Equation 3) $${\Delta G}_{c}={\Delta G}_{{\text{OH}}^{*}}-{\Delta G}_{O^{*}}-eU+{\Delta G}_{H^{+}}$$

(Equation 4) $${\Delta G}_{d}=-{\Delta G}_{{\text{OH}}^{*}}-eU+{\Delta G}_{H^{+}}$$

### Quantification and statistical analysis

1. Pre-processing of data: XRD, LSV, CV, and other electrochemical data were converted into TXT format by the corresponding instruments, and plotted by Origin software without normalization and evaluation of outliers. Raman data were preprocessed by baseline correction. The data of SEM, TEM, contact angle, and DFT calculations were in the form of pictures or numbers, which were directly drawn by PowerPoint software without conversion, normalization, and evaluation of outliers.
2. Data presentation: The electrochemical measurements were independently tested three times to avoid any incidental error. And the related error bars (presented in the form of mean ± SD) were also shown in the manuscript and supporting information.
3. Sample size for each statistical analysis: The sample size of the related electrochemical measurements was three.
4. Statistical methods used to assess significant differences with sufficient details: The statistical test was two-sided testing, the α value was 0.05, and related P values were analyzed by Student’s two-sided t-test and shown in the manuscript and supporting information.
5. Software used for statistical analysis: The related software was Origin and Powerpoint.
