# Supplementary material for: Precisely tuning the electronic states of organic polymer electrocatalysts via thiophene-based moieties for enhanced oxygen reduction reaction
Source: iScience. 2025 Feb 12;28(3):112007. doi: 10.1016/j.isci.2025.112007 (PMC11930369; doi:10.1016/j.isci.2025.112007)
Supplement: Document S1. Figures S1–S7 and Tables S1–S4 [file mmc1.pdf]

iScience, Volume *28*

Supplemental information

Precisely tuning the electronic states of organic polymer electrocatalysts via thiophene-based moieties for enhanced oxygen reduction reaction

Dongye Li, Binbin Wang, Kunpeng Zheng, Hongni Chen, Yali Xing, Yanzhi Xia, and Xiaojing Long

Supplemental information

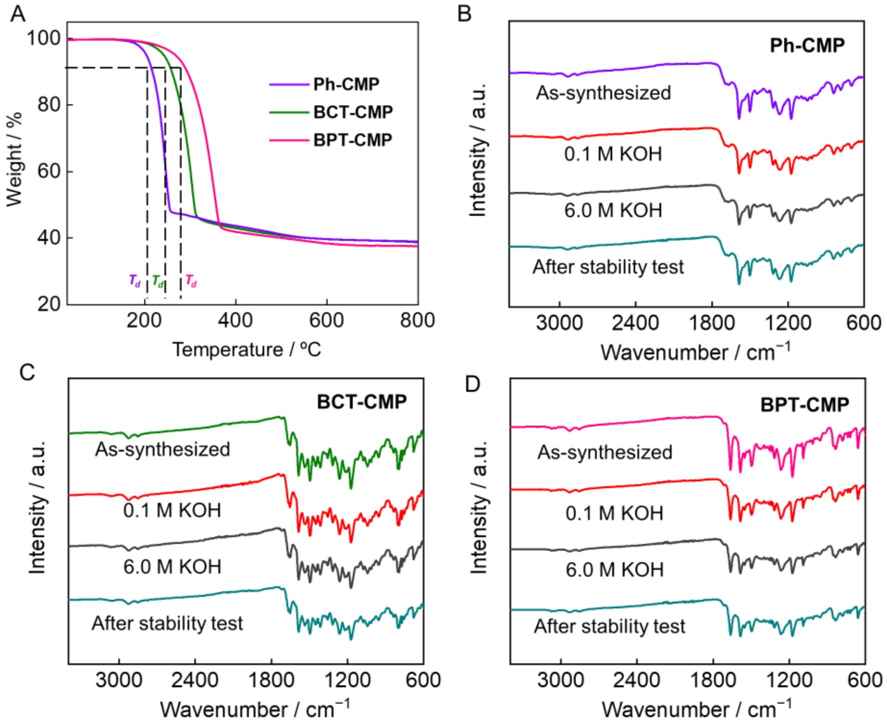

Figure S1. Stability test of Ph-CMP, BCT-CMP, and BPT-CMP.

(A) Thermogravimetric analysis (TGA) of **Ph-CMP**, **BCT-CMP** and **BPT-CMP**. (B) FT-IR of initial **Ph-CMP** and after alkaline treatment (under 0.1 M KOH and 6.0 M KOH for 24 hours) and stability test. (C) FT-IR of initial **BCT-CMP** and after alkaline treatment (under 0.1 M KOH and 6.0 M KOH for 24 hours) and stability test. (D) FT-IR of initial **BPT-CMP** and after alkaline treatment (under 0.1 M KOH and 6.0 M KOH for 24 hours) and stability test.

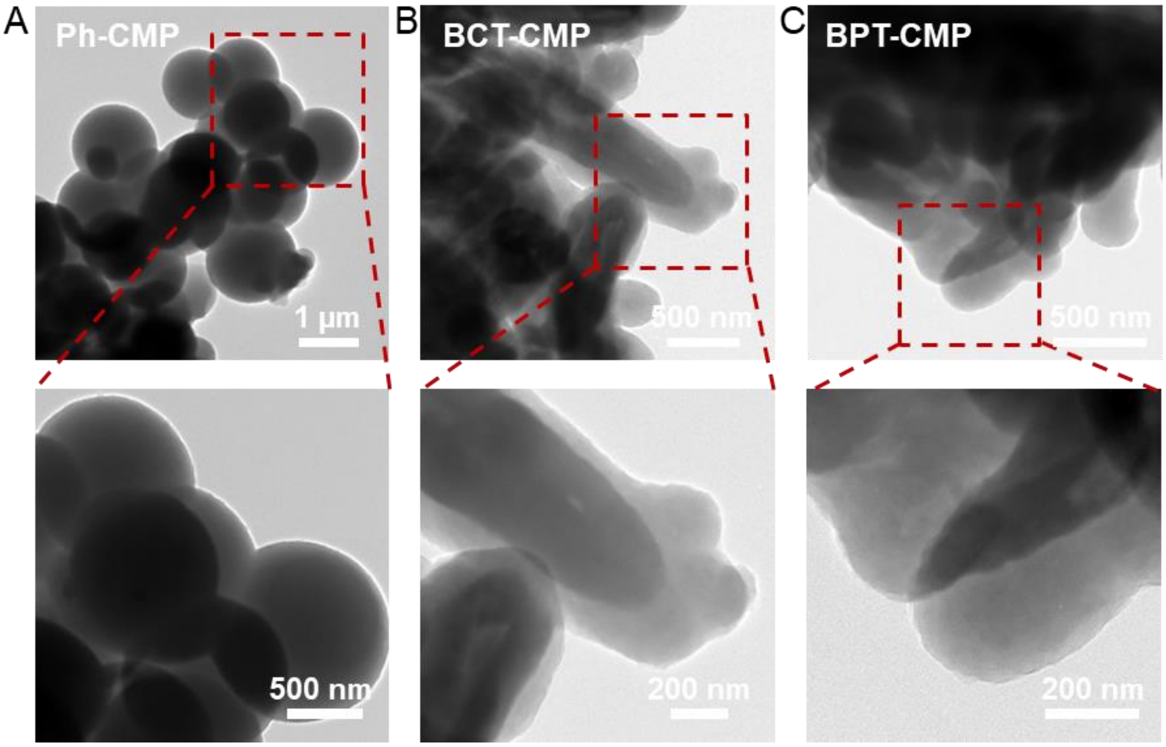

Figure S2. Morphological characterization.

TEM spectra of (A) **Ph-CMP**, (B) **BCT-CMP**, and (C) **BPT-CMP**.

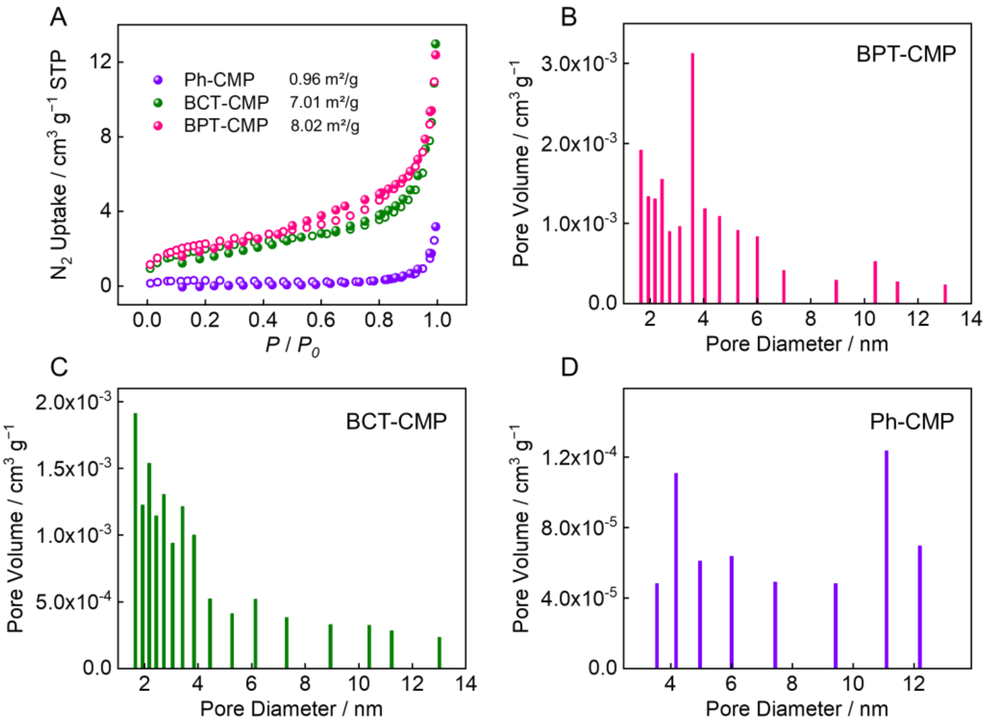

Figure S3. Brunauer-Emmett-Teller (BET) test of BPT-CMP, BCT-CMP, and Ph-CMP.

(A) N_2 adsorption isotherm (filled symbols) and desorption isotherm (open symbols) at 77 K. The pore size distribution curve of (B) **BPT-CMP**, (C) **BCT-CMP**, and (D) **Ph-CMP**.

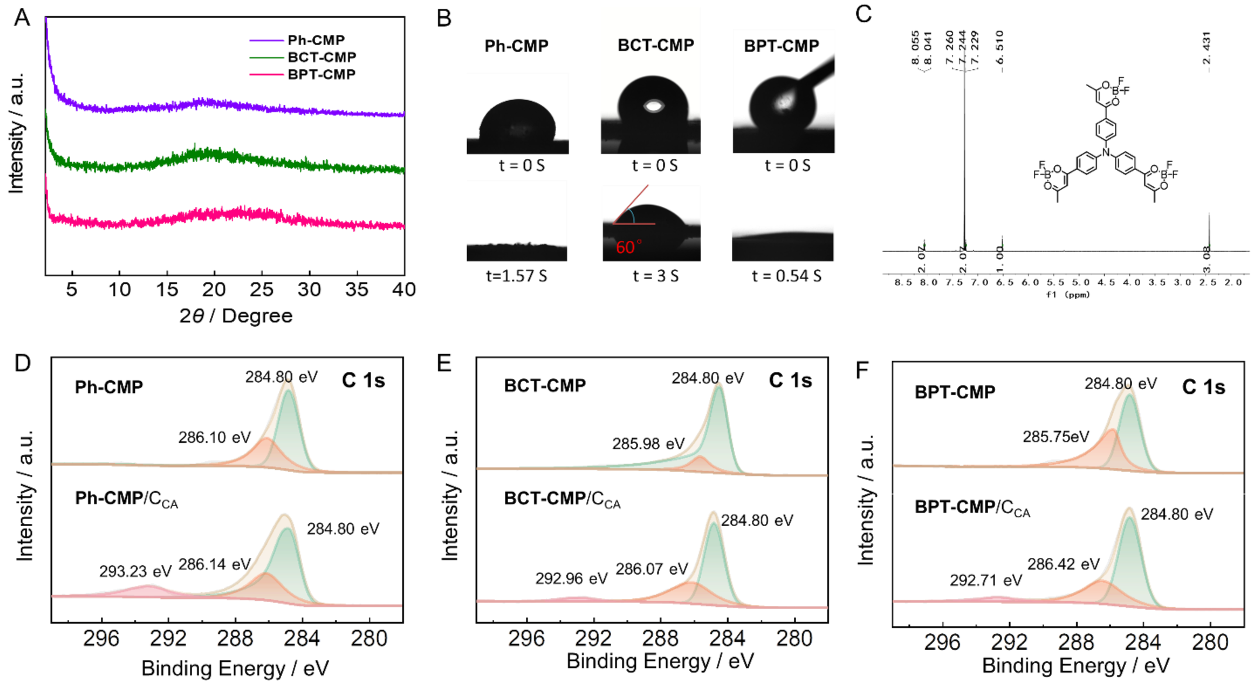

Figure S4. Structural characterization of Ph-CMP, BCT-CMP, and BPT-CMP.

(A) The PXRD patterns of **Ph-CMP**, **BCT-CMP**, and **BPT-CMP**. (B) Contact angle of **Ph-CMP**, **BCT-CMP** and **BPT-CMP**. (C) ^{1}H NMR spectrum of triphenylamine difluoroboronate monomer. (D) XPS spectra of C 1s and fitted **Ph-CMP** and **Ph-CMP**/C_{CA} results. (E) XPS spectra of C 1s and fitted **BCT-CMP** and **BCT-CMP**/C_{CA} results. (F) XPS spectra of C 1s and fitted **BPT-CMP** and **BPT-CMP**/C_{CA} results.

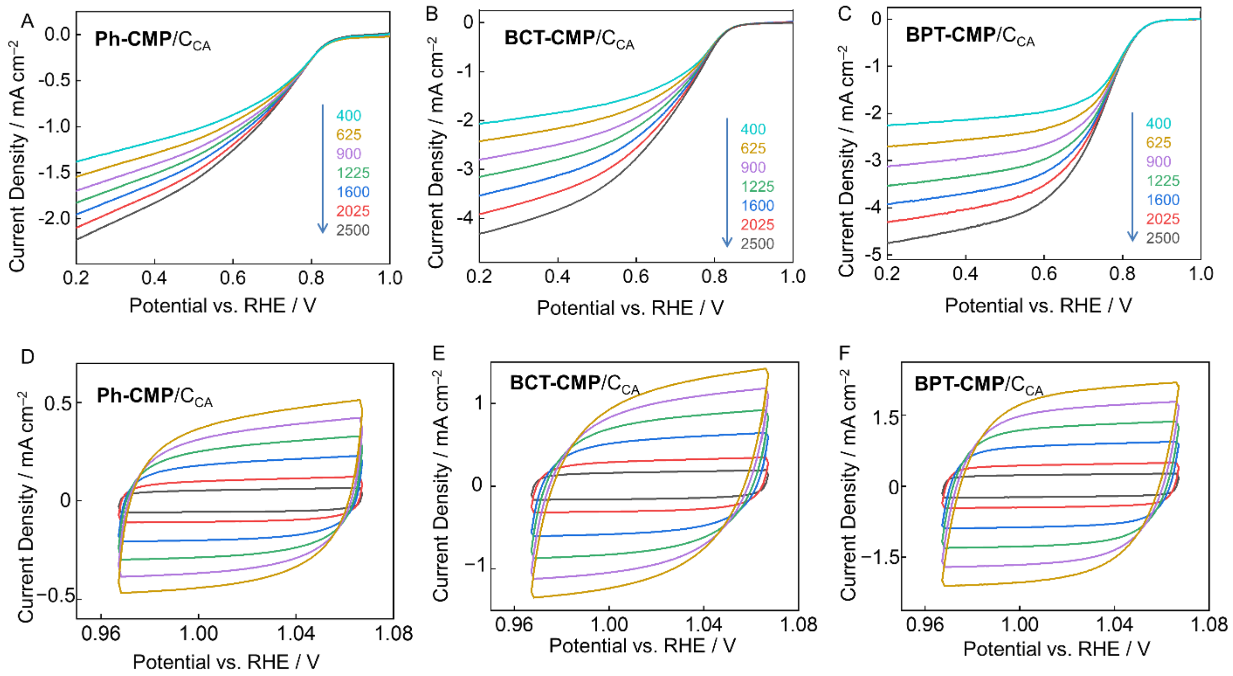

Figure S5. Electrochemical performance of Ph-CMP/C_{CA}, BCT-CMP/C_{CA}, and BPT-CMP/C_{CA}. LSV curves of (A) **Ph-CMP**/C_{CA}, (B) **BCT-CMP**/C_{CA}, and (C) **BPT-CMP**/C_{CA} from 400 to 2500 rpm. CV curves of (D) **Ph-CMP**/C_{CA}, (E) **BCT-CMP**/C_{CA}, and (F) **BPT-CMP**/C_{CA} with the scan rate from 10 mV s^{-1} to 100 mV s^{-1}.

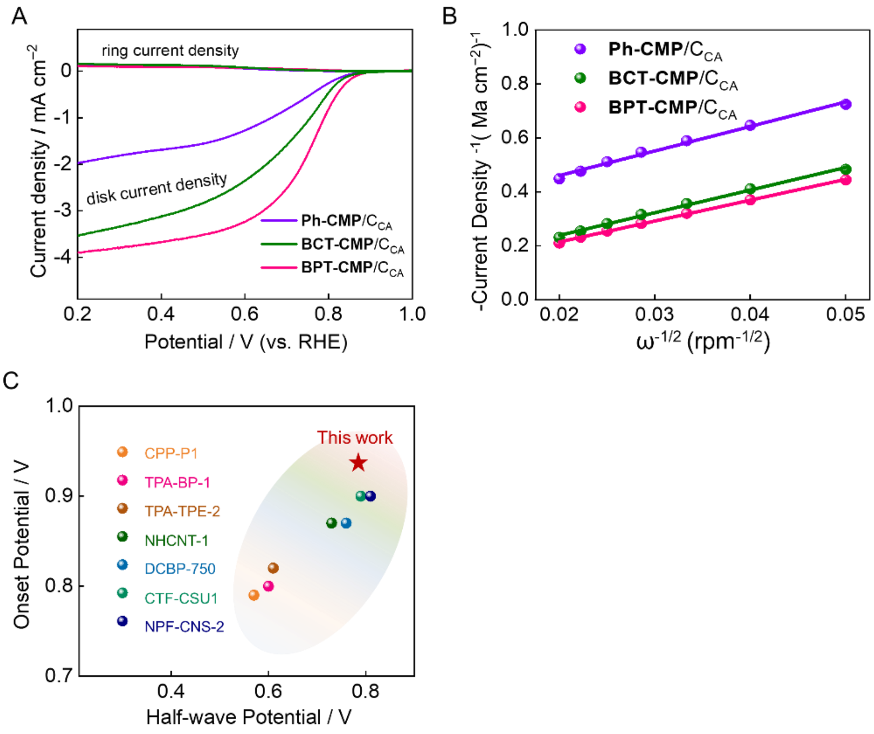

Figure S6. Electrocatalytic oxygen reduction reaction performance.

(A) The RRDE polarization curve of **Ph-CMP**/C_{CA}, **BCT-CMP**/C_{CA}, and **BPT-CMP**/C_{CA}. (B) The K-L plots for **Ph-CMP**/C_{CA}, **BCT-CMP**/C_{CA}, and **BPT-CMP**/C_{CA} at 0.2 V (vs RHE). (C) The onset and half-wave potential distributions of reported metal-free organic polymer catalysts and **BPT-CMP**/C_{CA} (this work).

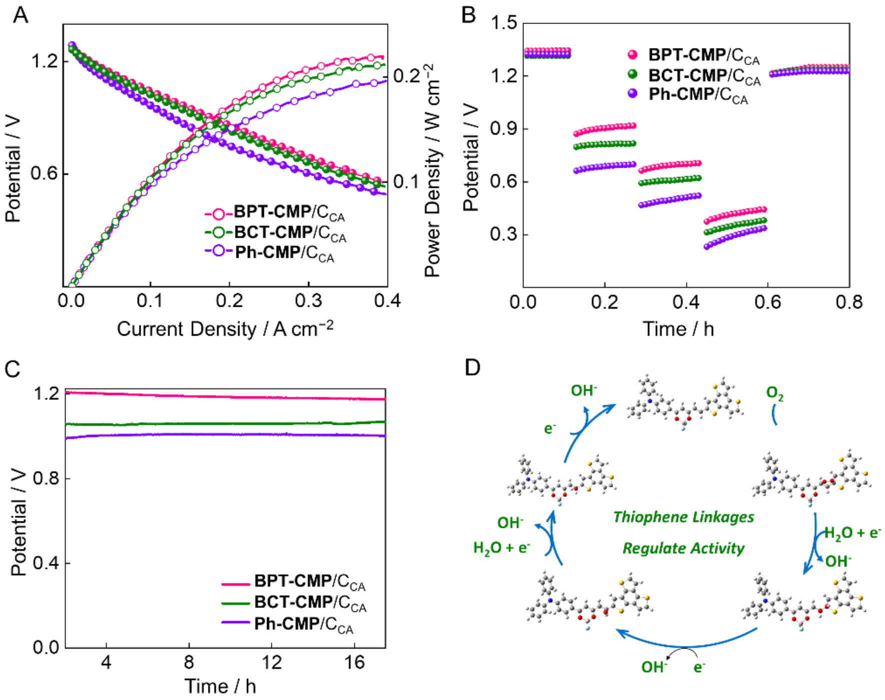

Figure S7. The zinc-air batteries (ZABs) performance and theoretical calculation.

(A) Discharge polarization curve and corresponding power density plot of **Ph-CMP**/C_{CA}, **BCT-CMP**/C_{CA}, and **BPT-CMP**/C_{CA}. (B) Discharge curves of **Ph-CMP**/C_{CA}, **BCT-CMP**/C_{CA}, and **BPT-CMP**/C_{CA} based ZABs at different current densities (25, 50, 100, 200 mA cm^{-2}). (C) Galvanostatic discharge curve of **Ph-CMP**/C_{CA}, **BCT-CMP**/C_{CA}, and **BPT-CMP**/C_{CA} based ZABs (6.0 M KOH electrolyte). (D) Theoretical ORR catalytic process of active site-3 in **BPT-CMP**.

Table S1. ORR performance of metal-free organic polymer catalysts.

Catalysts	E_{onset}(V vs RHE)	$E_{1/2}$(V vs RHE)	Eletrolyte	Ref.
TPA-BP-1	0.80	0.60	0.1 mol/L KOH	1
TPA-TPE-2	0.82	0.61	0.1 mol/L KOH	1
CPP-P1	0.87	0.73	0.1 mol/L KOH	2
NHCNT-1	0.87	0.76	0.1 mol/L KOH	3
CTF-CSU1	0.79	0.57	0.1 mol/L KOH	4
DCBP-750	0.90	0.79	0.1 mol/L KOH	5
NPF-CNS-2	0.90	0.81	0.1 mol/L KOH	6
BPT-CMP/C_{CA}	0.93	0.75	0.1 mol/L KOH	This work

Table S2. The free energy values of different sites in Ph-CMP.

Site	1	2	3
overpotential	1.05	0.86	1.02
ΔG1 (eV)	0.61	0.25	1.02
ΔG2 (eV)	−1.66	−0.09	−0.85
ΔG3 (eV)	1.05	0.86	0.42
ΔG4 (eV)	2E−4	−1.02	−0.58

Table S3. The free energy values of different sites in BCT-CMP.

Site	1	2	3	4	5	6
overpotential	1.03	1.53	0.61	0.94	0.65	1.04
ΔG1 (eV)	0.64	1.53	0.57	0.94	0.65	0.64
ΔG2 (eV)	−1.57	−1.54	−1.01	−1.39	−0.70	−0.44
ΔG3 (eV)	1.03	1.01	0.61	0.85	0.26	1.04
ΔG4 (eV)	−0.10	−1.00	−0.17	−0.40	−0.21	−1.24

Table S4. The free energy values of different sites in BPT-CMP.

Site	1	2	3	4	5	6
overpotential	1.02	1.11	0.58	0.88	1.09	1.19
ΔG1 (eV)	0.86	0.96	0.41	0.54	1.09	1.15
ΔG2 (eV)	−1.65	−1.6	−1.05	−0.16	−0.71	−1.09
ΔG3 (eV)	1.02	1.11	0.58	0.88	0.11	1.19
ΔG4 (eV)	−0.05	−0.47	0.06	−1.27	−0.49	−1.25

Supplementary references

[1] Roy, S., Bandyopadhyay, A., Das, M., Ray, P., Pati, S., and Maji, T. (2018). Redox-active and semi-conducting donor–acceptor conjugated microporous polymers as metal-free ORR catalysts. J. Mater. Chem. A 6, 5587. https://doi.org/10.1039/c8ta00099a.

[2] Bandyopadhyay, S., Boukhvalov, D., Nayak, A., Ha, S., Shin, H., Kwon, J., Song, T., and Choi, H. (2019). Redox active nitrogen-containing conjugated porous polymer: An organic heterogeneous electrocatalysts for oxygen reduction reaction. Dyes Pigm. 170, 107557. https://doi.org/10.1016/j.dyepig.2019.107557.

[3] Zhang, W., Sun, H., Zhu, Z., Jiao, R., Mu, P., Liang, W., and Li, A. (2019). N-doped hard carbon nanotubes derived from conjugated microporous polymer for electrocatalytic oxygen reduction reaction. Renew. Energy 146, 2270-2280. https://doi.org/10.1016/j.renene.2019.08.071.

[4] Yu, W., Gu, S., Fu, Y., Xiong, S., Pan, C., Liu, Y., and Yu, G. (2018). Carbazole-decorated covalent triazine frameworks: Novel nonmetal catalysts for carbon dioxide fixation and oxygen reduction reaction. J. Catal. 362, 1-9. https://doi.org/10.1016/j.jcat.2018.03.021.

[5] Sonmez, T., Belthle, K., Iemhoff, A., Uecker, J., Artz, J., Bisswanger, T., Stampfer, C., Hamzah, H., Nicolae, S., Titirici, M., and Palkovits, R. (2021). Metal free-covalent triazine frameworks as oxygen reduction reaction catalysts - structure-electrochemical activity relationship. Catal. Sci. Technol. 11, 6191. https://doi.org/10.1039/d1cy00405k.

[6] Zheng, Y., Song, H., Chen, S., Yu, X., Zhu, J., Xu, J., Zhang, K., Zhang, C., and Liu, T. (2020). Metal-Free Multi-Heteroatom-Doped Carbon Bifunctional Electrocatalysts Derived from a Covalent Triazine Polymer. Small 16, 202004342. https://doi.org/10.1002/smll.202004342.
